# Grating Structures for Silver-Based Surface Plasmon Resonance Sensors with Adjustable Excitation Angle

**DOI:** 10.3390/s24144538

**Published:** 2024-07-13

**Authors:** Pongsak Sarapukdee, Dirk Schulz, Stefan Palzer

**Affiliations:** Department of Electrical Engineering and Information Technology, Technical University Dortmund, Friedrich-Wöhler-Weg 4, 44227 Dortmund, Germany; pongsak.sarapukdee@tu-dortmund.de (P.S.);

**Keywords:** surface plasmon resonance (SPR), two-dimensional gratings, microfabrication, grating coupler, silver, sensors

## Abstract

Silver-based grating structures offer means for implementing low-cost, efficient grating couplers for use in surface plasmon resonance (SPR) sensors. One-dimensional grating structures with a fixed periodicity are confined to operate effectively within a single planar orientation. However, two-dimensional grating structures as well as grating structures with variable periodicity allow for the plasmon excitation angle to be seamlessly adjusted. This study demonstrates silver-based grating designs that allow for the plasmon excitation angle to be adjusted via rotation or beam position. The flexible angle adjustment opens up the possibility of developing SPR sensor designs with an expanded dynamic range and increased flexibility in sensing applications. The results demonstrate that efficient coupling into two diffraction orders is possible, which ultimately leads to an excitation angle range from 16° to 40° by rotating a single structure. The findings suggest a promising direction for the development of versatile and adaptable SPR sensing platforms with enhanced performance characteristics.

## 1. Introduction

The use of grating structures to excite surface plasmon resonances (SPRs) has a long-standing history [1] and is currently employed in various areas of sensing. Since the initial conceptual ideas, these structures have undergone extensive scrutiny, leveraging advancements in optical design to enable high surface sensitivity to detect changes in the surrounding medium [2] as well as allowing for integrated systems [3]. Presently, various avenues aim to enhance sensitivity, minimize structural dimensions, and explore alternative measurement methodologies [4,5]. The read-out technique when using grating structures [6,7,8] is often based on the angular modulation technique [5], in which the plasmon excitation angle before and after a sample reaction process is compared. Adjusting parameters such as the periodicity, grating thickness, and plasmonic materials enables the fine-tuning of the plasmon excitation angle. While this method produces reliable results, the plasmon excitation angle is fixed by the fabrication process of the grating, and only one specific angle [9,10] may be excited.

In order to enhance the dynamic range and flexibility of grating-based SPR sensors, more sophisticated grating designs including two-dimensional (2-D) gratings [11,12] and gradient grating periods (GGPs) [13,14] offer alternative operational modes for SPR sensor systems. Both approaches offer enhanced control over SPR excitation and tunability of the plasmon excitation angle without the need for changing the sensors system’s components.

The utilization of GGP and 2-D grating structures in SPR systems opens up new avenues for advanced sensing applications across various disciplines. In biosensing, these platforms offer improved detection limits [15,16], multiplexing capabilities [17], and enhanced selectivity [18] to molecular binding events. Moreover, the ability to tune [19,20] the plasmon excitation angle provides additional flexibility in optimizing sensing performance for diverse applications.

GGP structures rely on variations in the grating period along the surface, enabling tailored manipulation of plasmonic properties by changing the illuminated area. This flexibility allows for the optimization of SPR sensitivity and resolution for specific analytes and experimental conditions. Computer simulation studies have examined diffraction gratings with varying periods and found that coupled-mode equations accurately predict the behavior of compact varying-period gratings at oblique incidence using the Fourier modal approach [21]. The performance of the GGP structures is similar to conventional uniform grating designs if the gradient profile is tailored accordingly. Moreover, GGP structures offer the ability to tune the plasmon excitation angle, providing additional control over SPR response and enhancing the versatility of the sensing platform. GGPs have been previously examined in the context of guided-mode resonance (GMR) [22,23], and experimental realizations of GGP structures include a filter integrated into a portable spectrum detection device [24], covering a spectral range of 500–700 nm, and a guided-mode resonant filter (GMRF) with spectral reflectance wavelength changes based on the position of grating illumination [25].

Similarly, 2-D grating structures introduce additional degrees of freedom in SPR sensing, allowing for more precise tuning of plasmonic resonances and spatial distribution of electromagnetic fields [26]. To this end, hybrid structures combining metal films, dielectric films, and 2-D gratings for self-referenced refractive index sensing have already been examined [27]. Coupled-mode theory and finite-difference time-domain (FDTD) simulations underpin the potential for improved sensing performance and reliability using these structures. Among experimental realizations are a 2-D crossing grating for polarization-independent filters under oblique incidence [28] and a 2-D planar encoder using parallel gratings for multi-dimensional displacement measurements [29]. Metal-assisted guided-mode resonance (MAGMR) sensors in the visible spectrum range [30] have been demonstrated to outperform conventional GMR structures in sensitivity across various polarization modes. Lastly, a 2-D grating-based nanoscale precision interferometric measuring system has been studied [31]. Using a corner cube prism and a 2-D grating, this system allows for easy optical path modification and high-resolution measurements, with potential applications in accurate positioning and machining equipment. By manipulating both the periodicity and orientation of the grating structure in two dimensions, it can tailor SPR responses to specific analytes using the same grating structure. Furthermore, the 2-D nature of these structures enables tunability of the plasmon excitation angle, offering further control over SPR selectivity.

For applications in label-free, SPR-based analytics, the use of silver-based grating couplers is beneficial due to the possibly high sensitivity combined with low cost [32]. To this end, this study investigates the potential of silver-based structures for multiple plasmon excitation angles using a single-grating GGP structure and 2-D grating-based SPR systems, focusing on their ability to tune the plasmon excitation angle. The corresponding fabrication techniques and experimental methodologies are presented and discussed. The performance with respect to SPR angle tunability is characterized, highlighting the range of accessible resonances with a single grating structure. Additionally, the challenges and future prospects of these platforms are examined, aiming to provide insights into their continued development and widespread adoption in research and industrial settings.

## 2. Materials and Methods

**Figure 1:**
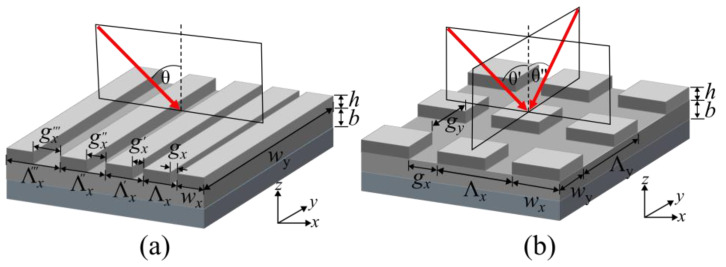
Schematic overview of the two silver-based grating structure approaches investigated for their performance. (**a**) A 1-D gradient grating period (GGP) structure allows for the excited SPR to be tuned via the position on the grating structure. (**b**) A 2-D grating structure allows for the SPR angle to be changed via rotation of the grating with respect to the exciting laser beam.

The fabrication of 2-D and GGP gratings is based on the established process described in [33]. In that study, the thickness and periodicity of the grating structure were thoroughly studied and optimized through extensive simulations and experiments. These optimized parameters are then adopted for use in the current work to ensure optimal performance of the sensor system. It features a 2 × 2 cm^2^ silicon (Si) wafer piece with a front side finish made of a 100 nm layer of thermally grown silicon oxide (SiO_2_). To facilitate adhesion of the silver (Ag) structures, 5–10 nm of nickel (Ni) is deposited prior to thermal evaporation of an Ag layer (Ag, 99.99% pure, Kurt J. Lesker Company Ltd., East Sussex, UK) to a thickness of 100 nm. Electron beam lithography using polymethyl-meth acrylate (PMMA; 4% 950 K, 679.04, Allresist GmbH, Strausberg, Germany) then defines grating structures with 2 different types of structure, i.e., 1-D gradient grating and 2-D grating structures. After developing the e-beam resist, a second silver deposition is performed to obtain the grating structure height (*h*) of 50 nm after a final lift-off process, resulting in the final structures as depicted in Figure 1.

The 1-D GGP structure is produced with a continuously changing gap length (*g_x_*) along the *x*-axis, known as a gradient grating period (GGP) [25,34], and a fixed 400 nm grating size (*w_x_*). The gap length g_x_ varies from 200 nm to 600 nm in increments of 1 nm every four periods, leading to an overall length of the structure of ~1.3 mm. This configuration results in a grating period (Λ) starting at 600 nm and concluding at 1000 nm along the grating area.

To investigate the performance parameters of 2-D gratings, symmetrical and non-symmetrical structures are produced. The latter structures feature consistent top and bottom spacings (*g* = 400 nm), equal width (*w* = 400 nm), and variable grating body lengths of 400 nm, 800 nm, 1600 nm, and 2000 nm, resulting in both square and rectangular shapes, as schematically shown in Figure 1b. The symmetric 2-D grating structures are designed with a square geometry, maintaining a constant period of 800 nm while introducing variations in the grating size. The utilization of e-beam lithography plays a crucial role in achieving this design, employing dose factor techniques to create a spectrum of grating sizes. This technique involves adjusting the dose of the electron beam (beam energy 10 keV, dose 55–70 μm/cm^2^), where higher energy doses lead to the formation of wider gratings.

The resulting structures are characterized using optical microscopy and scanning electron microscopy (SEM) in order to determine the structural parameters of the final devices. The coupling efficiency (CE) is calculated from the signal’s characteristics using the full width at half maximum (FWHM) of the relative reflectivity signal δR and the relative reflectivity $R_{rel}$ [33]:

$$CE=\frac{100-R_{rel}}{\delta R}$$

**Figure 2:**
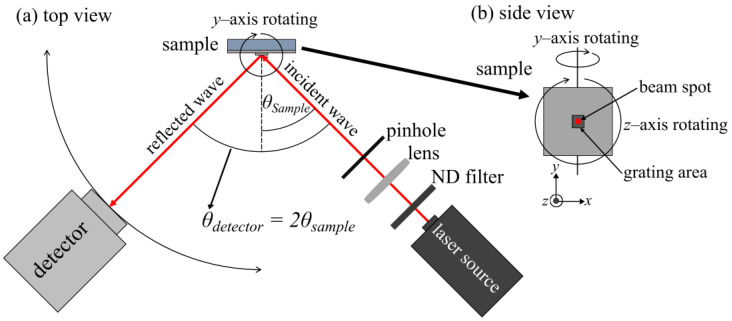
(**a**) The setup diagram displays the sample and detector aligned in the same plane, with the detector automatically rotating to capture reflections. Ensuring a 90° azimuthal angle, both the beam’s polarization and the grating structure can be adjusted. (**b**) In the side view of the sample, a grating area is located in the middle, with a beam spot inside it. The sample holder has the ability to rotate along the *y*- and *z*-axis.

To assess the performance of the grating structures in exciting surface plasmons, a modified version of the home-built measurement setup [33] is employed, as depicted in Figure 2. The helium–neon gas laser (05-LHP-151, Melles Griot, Singapore) emits light at 632.8 nm and is shaped into a Gaußian beam with 0.7 mm diameter. The sample holder allows for automatic recording of reflection characteristics from 10° to 40° with an angular resolution of 0.2° and rotation about the normal to the chip’s surface. The coupling efficiency is then evaluated by recording the reflected beam intensity for varying excitation angles with a light detector (ANDO, Ando Electric Co., Ltd., Tokyo, Japan) rotating simultaneously at twice the sample angle.

## 3. Results and Discussion

### 3.1. Asymmetric Two-Dimensional Grating Structures

**Figure 3:**
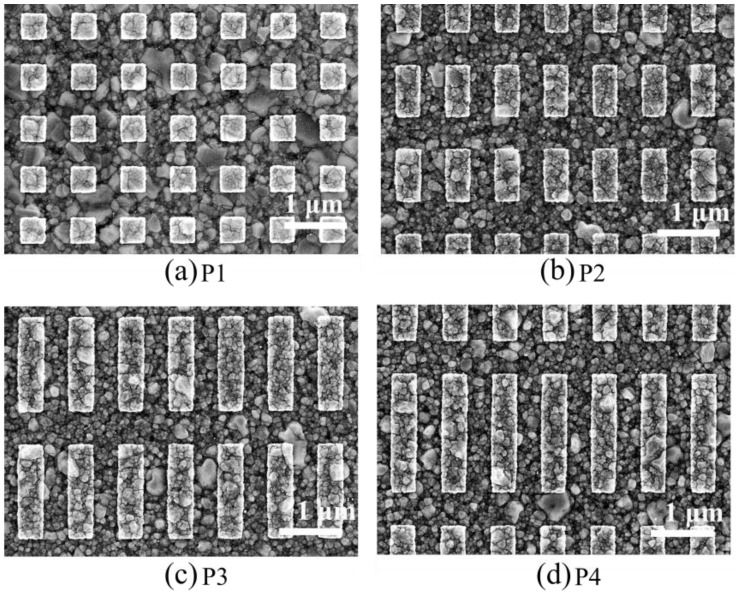
SEM images of the manufactured 2-D grating structure with width *w_y_* of 427 nm (**a**), 849 nm (**b**), 1544 nm (**c**), 1933 nm (**d**).

Two-dimensional (2-D) gratings are designed to have the same spacing with *g_x_*, *g_y_* = 400 nm and the same width with *w_x_* = 400 nm, and the length *w_y_* of the grating body is changed from *w_y_* = 400 to *w_y_* = 2000 nm, resulting in square and rectangular shapes, as exemplified in Figure 3.

**Figure 4:**
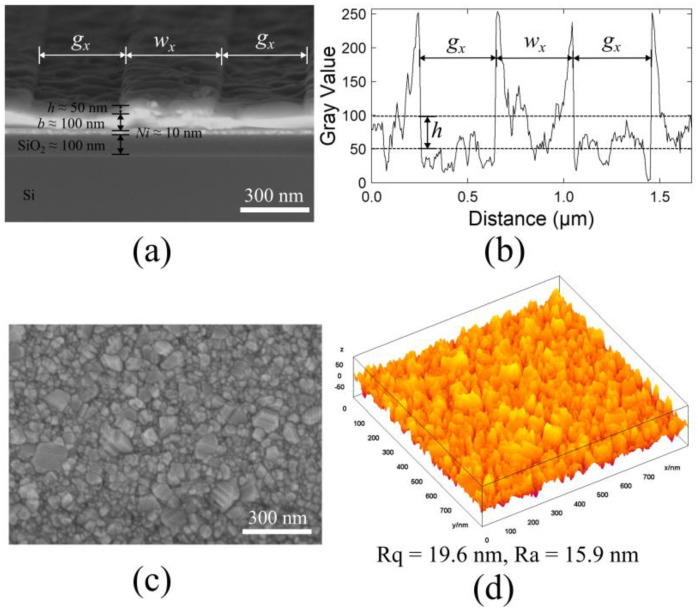
(**a**) SEM cross-section of the grating structure showing the dimensions *h*, *b*, *w_x_*, and *g_x_*. (**b**) Surface profile of the grating structure with a 400 nm width (*w_x_*) and a 400 nm gap (*g_x_*). (**c**) SEM image of the silver surface produced by thermal evaporation after lift-off process. (**d**) Surface roughness calculated from the SEM image using ImageJ with R_q_ = 19.6 nm and R_a_ = 15.9 nm.

Limitations in the fabrication process are assessed using scanning electron microscopy in order to confirm the actual grating sizes achieved, as well as the surface roughness, and Figure 4 illustrates the characterization of the grating structure. Figure 4a shows an SEM cross-section of the grating structure, highlighting key dimensions such as the height (*h*), base (*b*), width (*w_x_*), and gap (*g_x_*). The grating dimensions are measured at *w_y_* = 427 nm, 849 nm, 1544 nm, and 1933 nm, respectively, showcasing deviations from the originally intended sizes. Although these results do not align precisely with the initial expectations, the variations allow for the impact of these deviations on the overall performance of the gratings to be explored. Table 1 provides a comprehensive breakdown of the detailed sizes of the fabricated samples, highlighting the specific dimensions of each grating.

Figure 4b,c shows a representative surface profile of the grating structure, depicting the silver surface fabricated through thermal evaporation and the lift-off process. In particular, it showcases the texture and uniformity of the surface. The surface roughness is calculated from the SEM image using ImageJ software [35] with the roughness parameters R_q_ = 19.6 nm and R_a_ = 15.9 nm. All structures in the study are processed using the same standard procedure to ensure consistent structure morphology across samples. Consequently, the surface roughness of the different samples is considered unchanged. The surface brightness and roughness are estimated by analyzing SEM cross-sectional images, providing reliable data for the surface characteristics. This processing and the corresponding analysis confirm the reproducibility and reliability of the fabricated structures.

**Figure 5:**
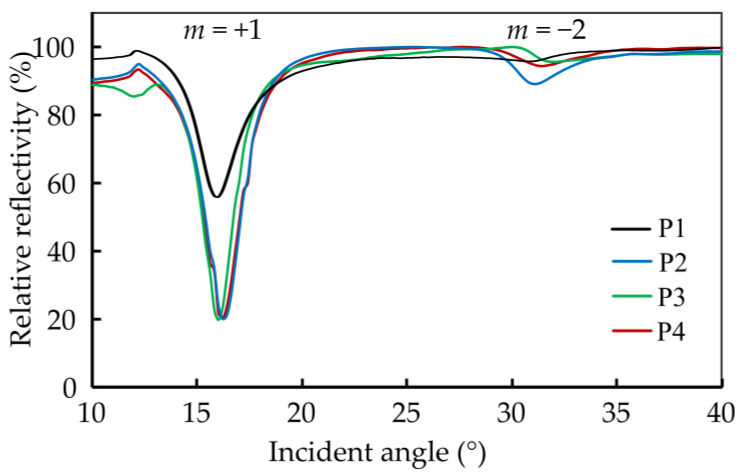
The relative reflection results show the plasmon excitation of 2-D grating of samples P1–P4. The coupling efficiency of P1 is considerably lower as compared to that of the other structures. P2 shows the highest coupling efficiency for the −2 order.

The behavior of the 2-D grating with variable width *w_y_* is analyzed using a plot of the light reflection against varying angles. Figure 5 represents the experimental outcomes, providing the relationship between grating width, plasmon excitation angles, and coupling efficiency as a function of *w_y_*.

The experimental findings reveal insights into the plasmonic behavior of the 2-D grating operated as a 1-D grating. Specifically, the maximum plasmon excitation angle is determined to be within the range of 16.0° to 16.2° (CE = 19.6–44.6) for the +1 diffraction order and 30.8° to 31.4° (CE = 0.8–3.6) for the −2 diffraction order. Notably, these outcomes are in line with those observed in previous studies involving silver-based 1-D gratings [33] in terms of the resonance angle. However, the coupling efficiency is critically dependent on the grating width *w_y_*. While for $w_{x}\;\geq$ 800 nm the coupling efficiency is in line with 1-D grating structures, it considerably diminishes for $w_{x}\;\approx \;w_{y}$.

The results indicate that the presence of a gap in the grating structure has no noticeable impact effect on the plasmon excitation angle for the +1 order for structures with a width $w_{y}\;\geq 2w_{x}$. In the case of the −2 order, the width ratio $\frac{w_{y}}{w_{x}}=2$ provides the best coupling results, a feature that cannot be observed in 1-D gratings.

### 3.2. Symmetric Two-Dimensional Grating Structure

**Figure 6:**
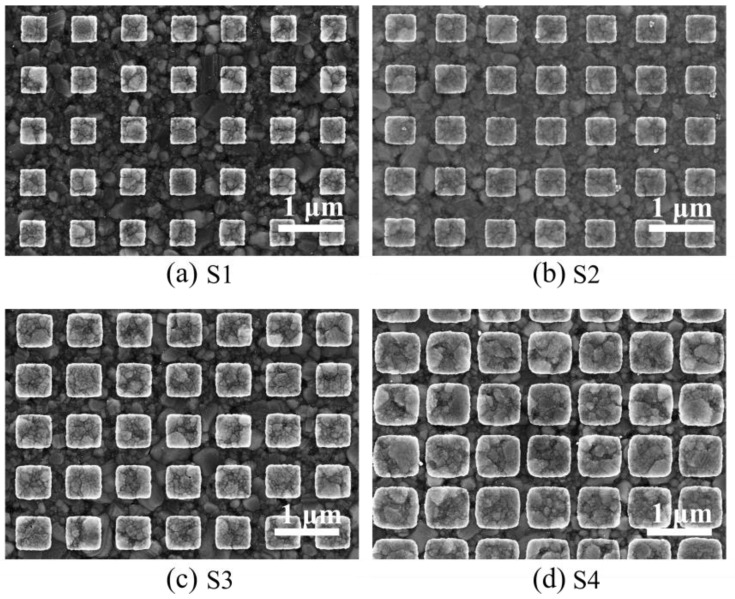
SEM images of grating structures with the same period (Λ = 800 nm) but different grating sizes (w) of 409 nm (**a**), 487 nm (**b**), 578 nm (**c**), and 718 nm (**d**) for samples S1, S2, S3, and S4, respectively.

The symmetric 2-D gratings utilize dose factor techniques during e-beam lithography to attain a diverse array of grating widths by employing different electron beam energies. The successful implementation of this approach is illustrated in Figure 6, where the symmetric 2-D grating is showcased for different grating sizes. Notably, the application of higher beam energy is instrumental in structuring larger grating sizes while maintaining a consistent period.

To assess the grating structure’s performance in a 1-D configuration, an analysis of the reflected light as a function of the incident angles is performed, starting with plotting the experimental data. The data show how variations in grating width impact on the optical response of symmetric 2-D gratings.

**Figure 7:**
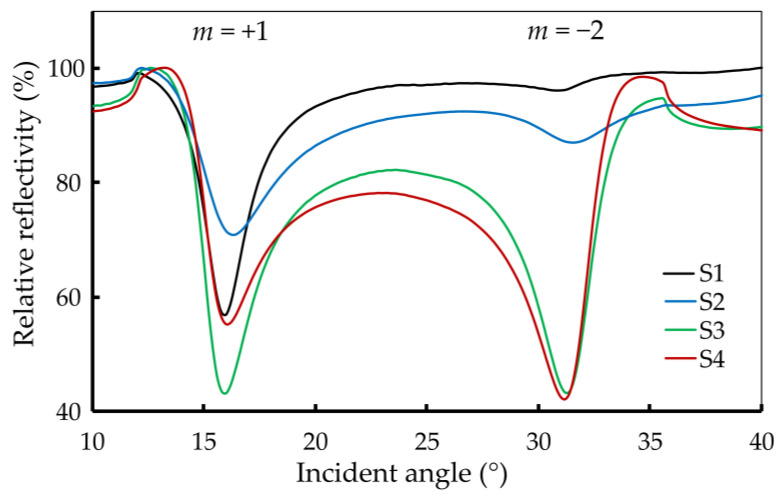
The relative reflection results for samples S1–S4 exhibit plasmon excitation at approximately 16.1° and 31.2° for the +1 and −2 orders, respectively.

Table 2 summarizes the grating parameters for grating structures S1–S4. The relative reflection signal profiles are displayed in Figure 7. The results indicate that the 2-D grating structures have a significant impact on the SPR characteristics, specifically in terms of excitation angles and coupling efficiencies. The controlled increase in grating periods and groove widths from S1 to S4 suggests a methodical approach to tuning the plasmonic properties. The plasmon excitation angles remain relatively stable for the +1 order but vary for the −2 order. Coupling efficiencies show a more complex behavior, with no direct correlation to the grating period and groove width alone.

Notably, the data highlight that an increase in the size of the grating tends to improve the CE for the −2 order. However, to maintain high efficiencies for both +1 and −2 orders, an optimal grating size is crucial. Sample S3 is identified as having the most favorable dimensions, achieving high CEs of 25.9 and 16.7 for the +1 and −2 orders, respectively. This suggests that careful tuning of grating dimensions is essential for optimizing SPR characteristics for various applications. This feature cannot be achieved with 1-D gratings, where the coupling efficiency for the −2 order is fixed by other parameters, such as grating height and silver base thickness. The observed coupling efficiency for the two orders is simultaneously at the same level, which has not been observed with 1-D gratings so far. The additional flexibility results in the possibility of efficiently using two diffraction orders using the same grating.

**Figure 8:**
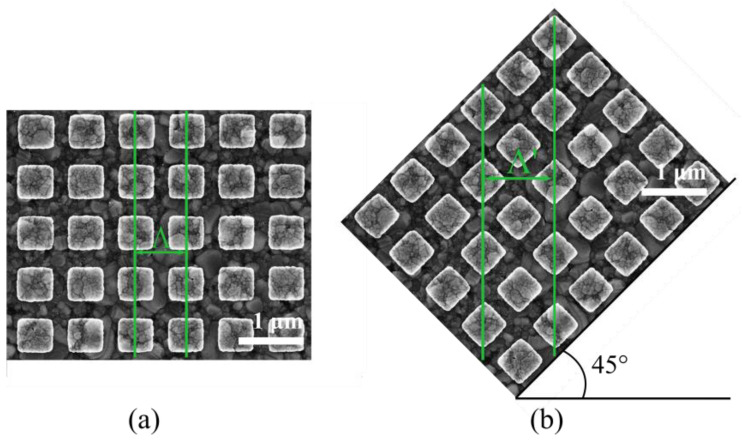
The grating at the position of 0° (**a**) has period size Λ and can represent the new grating arrangement with (**b**) a new period size Λ′ after the sample is rotated to 45°.

The plasmon excitation theory [36] explains the intricate dynamics using a grating structure and clarifies that, for effective excitation to occur, the direction of the incident electric field vector must align precisely with the orientation of the grating structure. Consequently, during the examination of the behavior of a 1-D grating structure, the sample cannot be rotated in order to maintain alignment between the incident light wave and the grating structure. However, the experimental approach shifts when dealing with 2-D gratings, which allows for the grating structure to be rotated, thus achieving different effective grating periodicies when the sample undergoes rotation in any direction. Figure 8 visually depicts the rotation of the sample during the experiments and the effect on the effective Λ. This enables the adaptation of the grating lines to align with incident light of any polarization, offering a more versatile and comprehensive exploration of plasmon excitation phenomena. To this end, the 2-D grating samples (S1–S4) are employed to investigate these distinctive properties.

**Figure 9:**
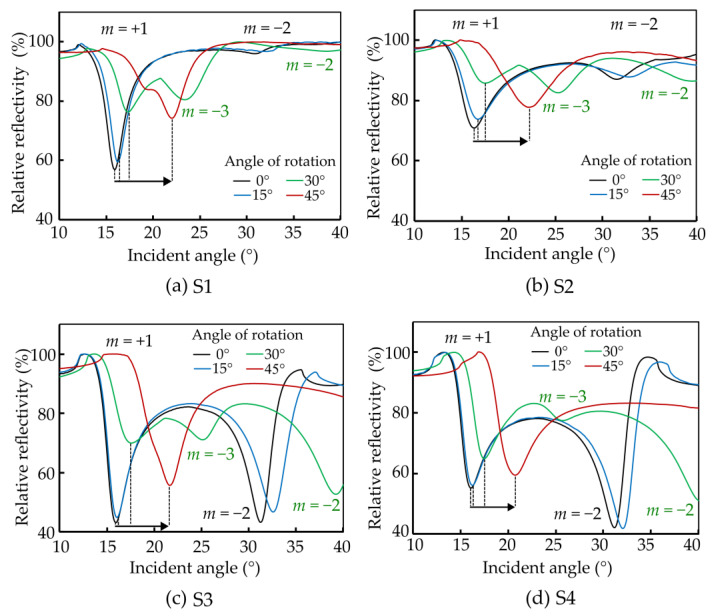
(**a**–**d**) (S1–S4) The image shows the sample rotation for 2-D grating measurement and the reflection properties of the 2-D grating coupler at 0–45° sample rotation angle. The results show that surface plasmon remains even as the sample rotates, which is not achievable with a 1-D grating. After rotation, the plasmon excitation angle of the structures is shifted to a higher incident angle for diffraction orders *m* = +1, *m* = −2, and *m* = −3. This shift indicates the robustness of the plasmonic response and highlights the advantage of using 2-D gratings for maintaining plasmon excitation under varying rotational conditions.

The characterization involves rotating the 2-D grating sample along the z-axis at various angles (0°, 15°, 30°, 45°), offering a quantitative insight into the impact of sample rotation on plasmon excitation characteristics. Figure 9 illustrates the surface plasmon excitation profile, providing a visual representation of the detailed interaction between the 2-D grating structure and incident light wave at different rotational angles.

The experiment’s results yield a notable finding, namely, that surface plasmon excitation continues to be efficient when the 2-D grating sample is rotated in different orientations. In sharp contrast, surface plasmon excitation is restricted by the fixed orientation of the grating lines in a 1-D grating. The results showcase a gradual increase in the excitation angle, rising from 16° at 0° rotation to 20.8° at a 45° rotation angle for the +1 order. At the same time, the CE is associated with the grating period since the coupling strength between light and surface plasmon at a specific wavelength scales with the grating vector [7].

Notably, the most substantial angle shift occurs at 45° rotation of the sample, surpassing the alterations observed at other rotational angles. The theoretical framework presented in [37] provides an explanation for this phenomena by outlining the rules governing grating excitations. The ensuing result analysis is presented in Table 3 for the two orders with highest CE. Nonetheless, with m = −3, a third type of order may be excited, as is visible in Figure 9. According to the theory [37] and corroborating prior research findings, key variables influencing the plasmon excitation angle are the effective periodicity and size of the grating. In line with this theoretical underpinning, the rotation of the sample introduces new grating periods, inducing a significant modification in the resultant plasmon excitation angle.

For each rotating angle, the effective grating’s period may be calculated for the +1, −2, and −3 diffraction orders, respectively. Notably, the grating period sizes generally increase with the rotation angle for symmetric 2-D gratings, reflecting the anticipated effect of rotation on the square grating structure’s interaction with the incident wave. For instance, at 0°, the grating periods range from 804 nm to 822 nm, whereas, at 45°, they extend from 903 nm to 941 nm for the +1 order.

The results also show that a single 2-D grating structure can be used to continuously tune the plasmon excitation angle between 16° and 40° using a rotation in combination with the two diffraction orders that show reasonably good coupling efficiency throughout the complete range, as shown in Figure 9. Interestingly, when the 2-D grating sample is rotated in this configuration, a dynamic shift in the surface plasmon excitation angle is observed. The measurement setup allows observation of incident angles up to 40°, which is why data for the −2 order at a 45° rotation are not observed. Consequently, the −2 order for a 45° rotation is unavailable. In contrast, a clear correlation between the angle of plasmon excitation and the angle of rotation is exhibited by the +1 diffraction order, which is significant as the +1 order possesses higher coupling efficiency than the −2 order, making it the most useful excitation angle for the sensors. For the 30° rotation, two apparently inconsistent sets of data are observed. However, the additional resonance is likely due to the −3 order.

**Figure 10:**
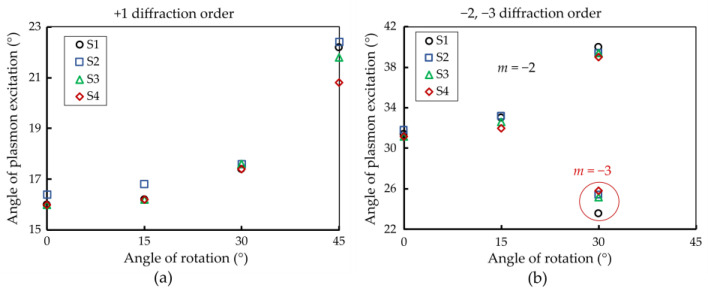
The relationship between the angle of rotation of a 2-D grating and the angle of plasmon excitation for +1 (**a**) and −2 (**b**) diffraction orders across four different samples (S1–S4). For both +1 and −2 orders, the angle of plasmon excitation increases consistently with the increasing angle of rotation of the 2-D grating. This indicates that the plasmon excitation angle for both orders is significantly affected by the grating rotation, demonstrating a similar dependency on the grating’s orientation.

Figure 10 illustrates the relationship between the plasmon excitation angle and the rotation angle of the structure for all observed diffraction orders, indicating a general increase in the plasmon excitation angle with larger rotation angles. At an angle of rotation of 30°, the m = −3 order is observed in addition, and, consequently, it is depicted in Figure 10b as well. The symmetry of the structure implies that, beyond 45° rotation, the structural configuration repeats, reverting to its initial form.

The adjustable angle feature of the 2-D grating structure can be utilized in biomedical diagnosis to facilitate double-check detection [38,39] for specific biomolecular interactions. When detecting low concentrations of biomolecules in complex biological samples, adjusting the excitation angle optimizes the signal-to-noise ratio [40], thereby improving detection limits and accuracy. This can be achieved through sequential measurement, where an initial measurement is performed using the first excitation angle, followed by a second measurement after rotating the sample. This method allows one device to obtain two datasets, which can be used to confirm the results. Alternatively, simultaneous measurement with two light beams and detectors oriented at different angles allows for real-time comparison and validation. Both methods ensure higher specificity, reduce false positives and negatives, and enhance the reliability of biomolecular detection.

### 3.3. Gradient Grating Structure

**Figure 11:**
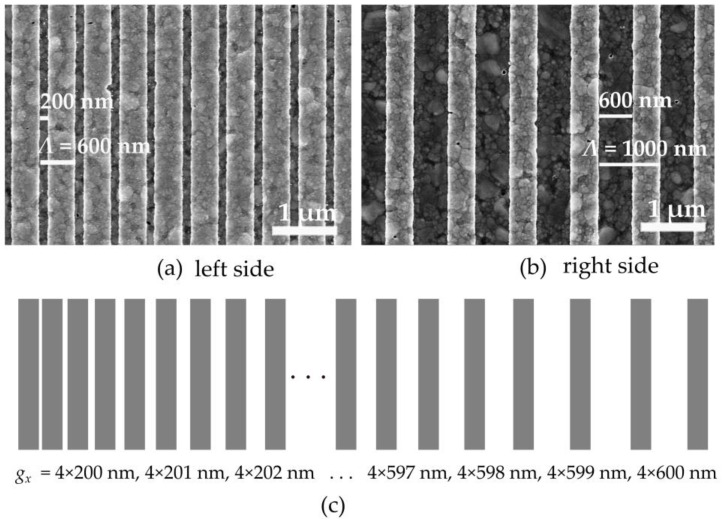
SEM images of a manufactured gradient grating with a shorter period (**a**) on the right and a longer period (**b**) on the left. (**c**) The design of the sample with different spot areas demonstrates that the gap changes along with the x-direction.

The 1-D gradient grating structures are fabricated with specific parameters to investigate their optical properties and maintain a consistent grating size (w) of 400 nm. However, the distance between the grids exhibits variability, ranging from 200 to 600 nm, with increments of 1 nm occurring every four grids. This design creates a grating period (Λ) that begins at 600 nm and ends at 1000 nm along the grating area, as shown in Figure 11. The entire grating area covers 1.3 mm^2^.

**Figure 12:**
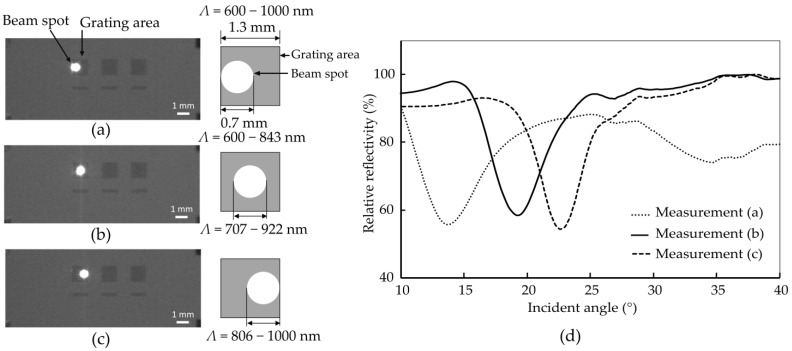
(**a**–**c**) Images showing three different grating areas of the sample with the beam spot positioned in various locations: (**a**) left side with a smaller gap (covered grating of Λ = 600–843 nm), (**b**) middle (covered grating of Λ = 707–922 nm), and (**c**) right side with a larger gap (covered grating of Λ = 806–1000 nm). The bright spot represents the beam location in each case. (**d**) The graph demonstrates the plasmon excitation angles obtained from different beam spot locations on the grating area, highlighting that one grating device can produce multiple plasmon excitation angles by altering the beam spot position.

To assess the reflectivity properties, the optical measurement system is used. However, it is crucial to note that the laser spot utilized for the optical measurements is strategically positioned at three distinct points within the grating area. The center of the laser beam is centered at 0.35 mm, 0.75 mm, and 0.95 mm from the edge corresponding to the side of the grating structure with a shorter grating period, as shown in Figure 12.

The measurement results show that, with the beam spot on the left side having the smallest gap, the plasmon excitation angle is 14.0° with a coupling efficiency (CE) of 6.5. Using the relation between plasmon excitation angle and constant grating period size from the referenced work [33], the calculated plasmon excitation angle for an average grating period size of 721 nm is 10.6°, while the measured angle is 14.0°. Similarly, with the beam spot in the center, the plasmon excitation angle is 19.4° with a CE of 8.9, and the calculated plasmon excitation angle is 16.5°. For the right side featuring the largest gap, the plasmon excitation angle is 22.8° with a CE of 13.2, and the calculated plasmon excitation angle is 20.8°. The experimental results are higher than the expected angles calculated using the average grating period size. These findings are summarized in Table 4, which shows the results of the plasmon excitation angle and coupling efficiency for the measurements (a–c).

These observations indicate that the plasmon excitation angle and coupling efficiency both increase as the beam spot moves from left to right, correlating with increasing gap sizes. There is a slight discrepancy between the calculated grating period and the averaged grating period sizes, suggesting that the gradient grating structure is more influenced by regions with bigger gaps. This is supported by the fact that the real experimental results consistently show larger plasmon excitation angles than those predicted for the same grating period size from a constant grating structure. The larger gaps likely contribute more to the effective coupling of the incident light to the plasmon modes, resulting in higher plasmon excitation angles and coupling efficiencies. This enhanced interaction in areas with larger gaps underscores the influence of the gradient grating design, where variable gap sizes create a more complex plasmonic response compared to uniform grating structures.

However, it is important to note that the efficiency of the gradient grating, with a relative reflection of about 55%, is observed to be lower compared to that of conventional structures. The process of designing and constructing a grating with multiple lengths proves to be challenging. Despite its limitations, this gradient grating approach introduces a novel dimension to grating design, offering a potential avenue for applications that prioritize adaptability over a uniform grating structure.

Gradient grating structures represent a significant advancement in the field of biosensors, offering unparalleled versatility and efficiency in biomolecule detection. Conventional biosensors often require dedicated design and fabrication processes for each target molecule, impeding rapid development and increasing costs. In contrast, gradient grating structures, with their unique ability to manipulate light through a varying refractive index and an adjustable excitation angle, create a flexible “all-in-one” platform [41]. By directing light at different grating periods, users can optimize the signal-to-noise ratio for a wide variety of biomolecules without needing constant redesigns. This adaptability is especially beneficial for detecting biomolecules with large binding-induced refractive index changes [42,43], accommodating a broader detection range through simple angle adjustments. As a result, gradient grating biosensors will accelerate research cycles and enhance productivity in biomedical diagnostics. Their capability to provide precise and reliable measurements across multiple applications underscores their potential to revolutionize biomolecule detection, paving the way for more efficient and cost-effective sensing solutions.

## 4. Conclusions

The experimental setups presented herein showcase the feasibility of constructing grating structures capable of efficiently generating multiple plasmon excitation angles from a single grating sample. Such versatility holds promise for a diverse range of applications, surpassing the adaptability of fixed-spacing gratings.

This study has demonstrated that surface plasmon polaritons can also be excited utilizing silver-based 2-D grating structures. Moreover, since these grating structures have more modifiable parameters than 1-D grating, they can be utilized more dynamically, depending on the applications. In particular, two diffraction orders may be excited with high coupling efficiency, offering the possibility to continuously access a range of plasmon excitation angles ranging from 16 to 40°. Two-dimensional gratings can exhibit anisotropic behavior, allowing for the independent measurement of strains or deformations along different axes. This capability is particularly valuable in applications such as strain sensing, where the ability to distinguish between axial and transverse strains is essential.

Both GGP and 2-D grating structures offer the potential for multi-parameter sensing capabilities compared to conventional 1-D gratings and traditional SPR sensing methods. These advanced grating designs open up new avenues for the development of high-performance, versatile, and miniaturized optical sensors for various applications in biosensing, environmental monitoring, and structural health monitoring.

## Figures and Tables

**Table 1:** Structural parameters of the grating structure produced and the relative reflection results show the plasmon excitation of 2-D grating of samples P1–P4.

Sample	Λ*_x_*/nm	Λ*_y_*/nm	*w_x_*/nm	*w_y_*/nm	Plasmon Excitation Angle (°), Coupling Efficiency	
					+1 Order	−2 Order
P1	807 ± 6	826 ± 8	409 ± 7	427 ± 6	16.0°, 19.6	30.8°, 0.9
P2	808 ± 6	1373 ± 4	412 ± 8	849 ± 7	16.2°, 36.4	31.0°, 3.6
P3	811 ± 6	2066 ± 5	426 ± 6	1544 ± 6	16.0°, 44.6	31.4°, 0.8
P4	811 ± 7	2451 ± 5	409 ± 8	1933 ± 7	16.2°, 36.3	31.4°, 1.7

**Table 2:** Structural parameters of the grating structure produced. The standard deviation of the periods is stated.

Sample	Λ*_x_*/nm	Λ*_y_*/nm	*w_x_*/nm	*w_y_*/nm	Plasmon Excitation Angle (°), Coupling Efficiency	
					+1 Order	−2 Order
S1	807 ± 6	826 ± 8	409 ± 7	427 ± 6	16.0°, 19.6	30.8°, 0.9
S2	811 ± 6	828 ± 5	487 ± 4	458 ± 5	16.0°, 6.1	31.2°, 5.2
S3	816 ± 4	819 ± 5	578 ± 5	551 ± 4	16.4°, 25.9	31.6°, 16.7
S4	821 ± 10	837 ± 2	718 ± 7	689 ± 7	16.0°, 6.9	31.2°, 12.9

**Table 3:** Results of plasmon excitation angle and coupling efficiency of the samples S1 to S4 with rotation angle of 10–45°. The plasmon excitation angles are presented for the +1 and −2 diffraction orders. At 30° rotation angle, the m = −3 order is observed, and the analysis is included in this summary.

Rotation Angle (°)	Plasmon Excitation Angle (°), Coupling Efficiency (CE)							
	S1		S2		S3		S4	
	+1 Order	−2 Order	+1 Order	−2 Order	+1 Order	−2 Order	+1 Order	−2 Order
0	16.0°, 19.6	31.2°, 0.9	16.4°, 5.9	31.8°, 3.4	16.0°, 25.9	31.2°, 15.8	16.0°, 7.2	31.2°, 12.9
15	16.2°, 18.5	32.0°, 8.9	16.8°, 5.0	33.4°, 2.6	16.2°, 25.1	32.6°, 13.0	16.4°, 7.0	32.0°, 12.9
30	17.4°, 5.7	38.6°, 0.4	17.4°, 2.3	40°, 1.9	17.4°, 3.9	39.2°, 9.1	17.6°, 6.1	40.0°, 9.1
		* 23.2°, 3.3		* 25.4°, 4.0		* 25.2°, 6.8		* 25.8°, 5.2
45	20.8°, 5.2	NA, NA	22.4°, 3.7	NA, NA	21.8°, 9.4	NA, NA	20.8°, 6.2	NA, NA

* −3 diffraction order.

**Table 4:** Results of plasmon excitation angle and coupling efficiency of the measurements (a–c).

Measurement	Grating Period Size (nm)	Plasmon Excitation Angle (°), Coupling Efficiency (CE)	Averaged Grating Period Size (nm)	Plasmon Excitation Angle (°) (Theory)
(a)	600–843	14.0°, 6.5	721	10.6°
(b)	707–922	19.4°, 8.9	815	16.5°
(c)	806–1000	22.8°, 13.2	903	20.8°

## Data Availability

The data underlying the results presented in this paper are not publicly available at this time but may be obtained from the authors upon reasonable request.
